# Guillain-Barré syndrome involving reproductive system revealed on ^18^F-FDG PET/CT: a case report

**DOI:** 10.3389/fimmu.2026.1811782

**Published:** 2026-06-01

**Authors:** Huiling Li, Xinzhong Hao, Zhifang Wu, Sijin Li, Li Li

**Affiliations:** 1 Department of Nuclear Medicine, First Hospital of Shanxi Medical University, Taiyuan, Shanxi, China; 2 Collaborative Innovation Center for Molecular Imaging of Precision Medicine, Shanxi Medical University, Taiyuan, Shanxi, China

**Keywords:** ^18^F-FDG PET/CT, Guillain-Barré syndrome, orchitis, prostatitis, radiculitis

## Abstract

Guillain-Barré syndrome (GBS) is an immune-mediated acute inflammatory peripheral neuropathy. While it is well-established that its typical manifestations involve the peripheral nerves, it is noteworthy that involvement of the central nervous system and the reproductive system is exceedingly rare. Here we report the case of a 67-year-old man with GBS in whom the ^18^F-FDG PET/CT demonstrated systemic inflammatory hypermetabolism. The imaging revealed multifocal, heterogeneous high-activity along the nerve roots, cerebral cortex, hypothalamus, and medulla oblongata. Crucially, it also identified significant involvement of the right testis and the transition zone of the right prostate, suggesting direct inflammatory engagement of the reproductive system. This case underscores the potential of ^18^F-FDG PET/CT as a comprehensive imaging tool for assessing the extent of systemic disease and inflammatory activity in complex GBS presentations.

## 1 Introduction

Guillain-Barré syndrome (GBS) is an immune-mediated acute inflammatory peripheral neuropathy characterized by rapidly progressive flaccid paralysis. Involvement of the central nervous system (CNS) and the reproductive system is uncommon and has been described in only a small number of case reports. Scoppetta et al. (1) reported two cases with CNS involvement. However, reports on CNS and reproductive system involvement remain scarce, and large-scale clinical studies are lacking. Here we report the case of a 67-year-old man with GBS in whom the ^18^F-FDG PET/CT revealed multifocal, heterogeneous hypermetabolism along the nerve roots, cerebral cortex, hypothalamus, and medulla oblongata. Crucially, it also identified significant involvement of the right testis and the transition zone of the right prostate.

## 2 Case description

A 67-year-old man presented with right facial paralysis, progressive dysphagia, and bilateral lower limb weakness persisting for over one week, accompanied by decreased sensation in the lower limbs. One month after admission, the patient suddenly experienced respiratory arrest and underwent emergency tracheal intubation for assisted ventilation. Electromyography indicated extensive damage to the peripheral nerves of the limbs, affecting both sensory and motor functions. Cerebrospinal fluid examination revealed protein-cell dissociation. Based on the acute onset of progressive flaccid paralysis, along with the electrophysiological evidence of peripheral nerve involvement and the characteristic CSF finding of protein-cell dissociation, a preliminary diagnosis of GBS was established.

**Figure 1:**
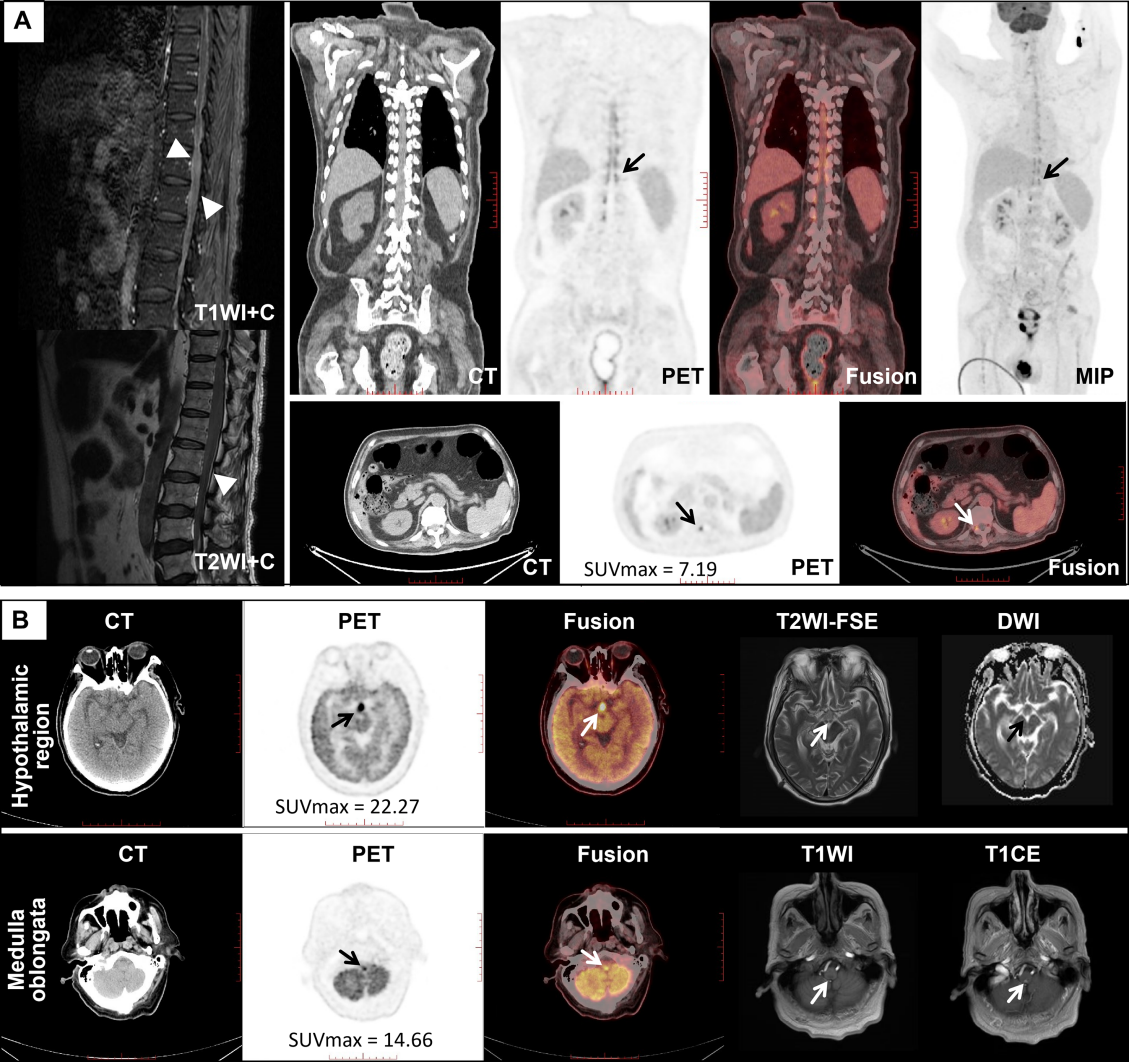
Imaging features of GBS with nervous system involvement. **(A)** Typical polyradiculitis: Enhanced MRI shows abnormal enhanced foci within the spinal canal at the T11-L2 level, associated with spinal cord compression and edema (white triangles). PET/CT fusion images demonstrate increased FDG uptake in the nerve roots of the cervical, thoracic, and lumbar segments of the spinal cord (arrows); **(B)** atypical CNS involvement: multiple diffuse and heterogeneous areas of increased FDG metabolism are observed on the surface of the cerebral cortex, cerebellum, hypothalamic region (upper row, arrows), and dorsal medulla oblongata (lower row, arrows), indicating a widespread inflammatory process extending beyond the typical lesion range. The enhanced MRI of the aforementioned area shows abnormal signals (arrows).

**Figure 2:**
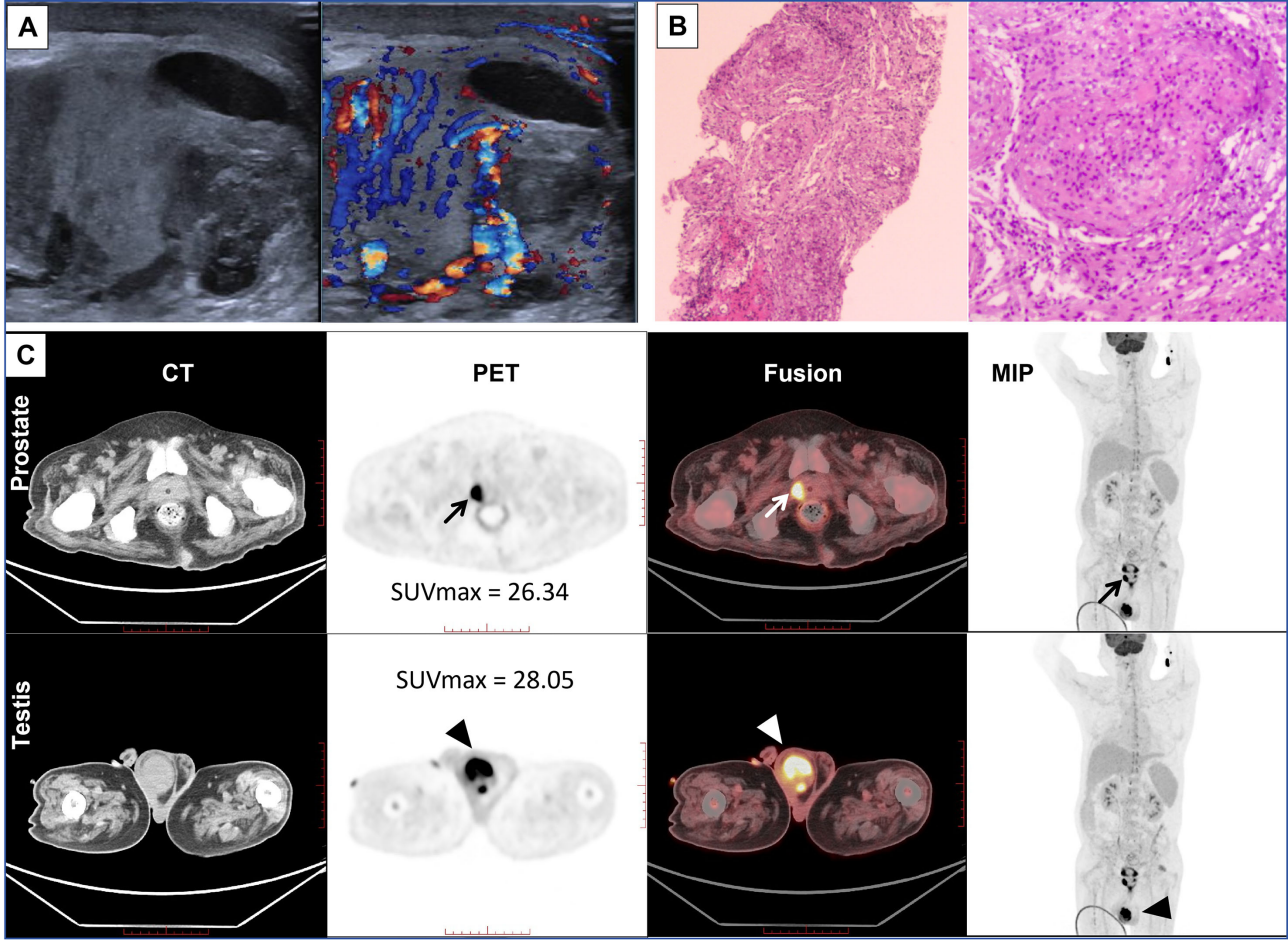
Imaging manifestations of the reproductive system. **(A)** color doppler ultrasound reveals diffuse scrotal wall thickening and inflammatory changes involving the right epididymis, testis, and spermatic cord, accompanied by epididymal liquefaction; **(B)** histopathological examination of the testicular lesion demonstrates the presence of inflammatory infiltration; **(C)** ^18^F-FDG PET/CT shows corresponding intense hypermetabolism in the right prostate (upper row, arrows) and right testis (lower row, triangles).

Further investigations were performed to assess the extent of disease involvement. The enhanced MRI (**Figure 1A**) showed abnormal enhanced foci within the spinal canal at the T11-L2 level, accompanied by spinal cord compression and edema. The patient also presented with urinary symptoms such as difficulty urinating. Color Doppler ultrasound (**Figure 2A**) showed diffuse scrotal wall thickening and inflammatory changes of the right epididymis, testis, and spermatic cord, accompanied by epididymal liquefaction.

In view of the patient’s unexplained systemic inflammatory response and neurological symptoms, ^18^F-FDG PET/CT was performed to rule out occult tumors or infectious foci. PET/CT fusion images (**Figure 1A**) showed increased FDG uptake in the nerve roots of the cervical, thoracic and lumbar segments of the spinal cord in PET/CT fusion images. Multiple diffuse and heterogeneous increased FDG metabolism were observed on the surface of the cerebral cortex, cerebellum, hypothalamic region and the dorsal part of the medulla oblongata (**Figure 1B**), indicating a widespread inflammatory process beyond the typical lesion range. FDG PET/CT also demonstrated intense hypermetabolism in the right prostate and right testis (**Figure 2C**). Histopathological examination of the testicular lesion revealed inflammatory infiltration (**Figure 2B**), further immunohistochemical characterization showed a mixed T-cell (CD3+) and B-cell (CD20+) infiltrate, with partial co-expression of CD5 and the presence of plasma cells (CD138+, CD38+). A Ki-67 index of 20% was noted. These findings confirmed the diagnosis of orchitis. After receiving the standardized treatment with intravenous immunoglobulin (IVIG), his symptoms have improved significantly.

## 3 Discussion

GBS is a rare but potentially life-threatening immune-mediated polyneuropathy, with an estimated annual incidence of 1.12 per 100,000 population (2, 3). Its typical symptoms include abnormal sensations, pain, or weakness in the limbs, and the condition worsens rapidly within a few days (4). Severe cases may develop quadriplegia and require mechanical ventilation due to respiratory failure (5). GBS may also affect cranial nerves causing facial paralysis, ophthalmoplegia, or bulbar weakness. Nevertheless, over the past decades, the phenotypic spectrum of GBS has expanded considerably to include various atypical features (6). The present case further expands this spectrum by demonstrating extraneural involvement of the reproductive system and CNS visualized by ^18^F-FDG PET/CT.

Due to the diversity of clinical manifestations, early diagnosis can be challenging (7). Cerebrospinal fluid (CSF) analysis, including cell counts and protein levels, is recommended to rule out other diseases. The characteristic manifestation of CSF in GBS patients is protein-cell dissociation, with elevated protein levels (> 0.45 g/L) and a normal white blood cell count (typically < 5 cells/μL) (8). The typical protein-cell dissociation observed in this patient provided crucial support for the diagnosis of GBS. Subsequent electromyography confirmed peripheral nerve involvement (9).

However, the case challenges the traditional perception that GBS primarily involves peripheral nerves (7). What truly distinguishes this case from all previous reports of GBS is the unexpected discovery through ^18^F-FDG PET/CT. To rule out potential malignant tumors, which is a routine screening for atypical neurological manifestations, the patient underwent an FDG PET/CT examination. The increased FDG uptake of spinal nerve roots at multiple segments is consistent with the imaging features of GBS. Although the hypermetabolism of the cerebral cortex, hypothalamus and medulla oblongata observed by PET/CT does not represent a typical manifestations of GBS and needed to be differentiated from neurolymphoma, common causes such as tumors were ruled out after a comprehensive differential diagnosis. The right prostate and right testis exhibited increased FDG uptake with maximum standardized uptake values (SUVmax) of 26.34 and 28.05. This imaging finding has never been previously reported in the literature on GBS.

How can this phenomenon be explained? We propose an explanation based on the “molecular mimicry” hypothesis. The classical pathogenesis of GBS involves molecular mimicry, in which lipopolysaccharide from infectious agents, such as Campylobacter jejuni, triggers a cross-immune reaction against the gangliosides on the peripheral nerves (eg. GM1, GD1a) (10). Although GBS mainly affects the peripheral nerves, CNS involvement has been occasionally reported in variant forms or severe GBS (1). Autoantibodies or activated T cells may target regions within the CNS that express similar ganglioside antigens by crossing a damaged blood-brain barrier, presenting as an “immune encephalitis”-like process. This mechanism is similar to that observed in GBS overlap syndrome, such as Bickerstaff brainstem encephalitis (11).

At the same time, the reproductive system, as an immunologically privileged site, may also experience barrier disruption through during intense immune responses, leading to inflammation (12, 13). It is worth noting that previous studies have confirmed that prostate epithelial cells also express various gangliosides, including GM1 and GD1a (14). Based on pathological and immunohistochemical verification of the testicular lesion, we speculate that in this patient, activated autoimmune T cells or antibodies not only attacked the myelin sheath of the peripheral nerves, but also cross-reacted with ganglioside antigens co-expressed on the surface of prostate epithelial cells, leading to local immune activation, thereby manifesting as high metabolism on FDG PET/CT.

Based on the autoimmune mechanism of GBS, we hypothesize that a broad non-infectious inflammatory response may simultaneously attack the peripheral nervous system, the CNS and the reproductive system. However, this association still requires further validation through more cases. ^18^F-FDG PET/CT imaging, as an exclusive diagnostic method, is well-established imaging modality for detecting lesions with increased glucose metabolism. The present case highlights the remarkable sensitivity of FDG in detecting organs affected by GBS throughout the body, thus enabling a more comprehensive, one-stop evaluation. This outstanding diagnostic ability makes ^18^F-FDG PET/CT a valuable diagnostic tool for the auxiliary diagnosis, activity assessment, and treatment guidance of GBS. It should be noted that ^18^F-FDG PET/CT findings of neurolymphomatosis (NL) may mimic those observed in GBS, as both conditions can involve increased FDG uptake along peripheral nerves, potentially leading to diagnostic confusion. NL typically presents as linear or nodular hypermetabolism along the nerve tracts, with an asymmetric and multifocal distribution and most patients have a history of lymphoma (15). In contrast, FDG uptake in GBS is relatively mild and often shows a symmetrical and diffuse pattern of nerve involvement, Patients usually have a history of preceding infection. These differences in metabolic intensity, involvement pattern, and clinical context are key to their differentiation. In the present case, the diagnosis of GBS was supported by progressive limb weakness, protein-cell dissociation in the CSF, symmetrical and diffuse neurological involvement on FDG PET/CT, and the absence of a history of lymphoma.

The therapeutic response further supports the above inference of an inflammatory mechanism. IVIG and plasma exchange (PEX) are effective for the acute treatment of GBS and can prevent further immune-mediated damage to peripheral nerves (16). In the present case, the patient’s clinical symptoms significantly improved after receiving standardized gamma globulin infusion therapy, which indirectly confirms that the abnormally high uptake shown by PET/CT is attributable to reversible autoimmune inflammation rather than tumors or infections.

The limitation of this case is that, as a single case, it cannot determine the actual incidence of prostate and CNS involvement in the GBS population. Nevertheless, this case holds significant clinical and scientific value: it provides the first functional imaging evidence indicating that the autoimmune response of GBS is not limited to peripheral nerves but may also involve extraneural organs (such as the prostate) and the CNS that express common antigens.

## 4 Conclusion

This case expands the clinical phenotypic spectrum of GBS and reports the unexpected discovery of extraneural and CNS involvement in GBS by ^18^F-FDG PET/CT for the first time. We highlight the value of PET/CT in revealing the systemic inflammatory burden of GBS and suggest that for complex cases, the possibility of multi-system involvement extending beyond the typical boundaries should be considered.

## Data Availability

The original contributions presented in the study are included in the article. Further inquiries can be directed to the corresponding author.

## References

[1] Scoppetta C Fontana M Quadrini R La Cesa I Di Lello R Peppe A . Miller fisher syndrome: review of the literature and presentation of 2 cases. Riv Neurol. (1991) 61:137–44. 1667714

[2] Hidig MFO Sheikh Hassan M Ibrahim AA Adam BA Sidow NO Mohamed SA . Paralytic ileus as the initial presentation of Guillain-Barre syndrome: a rare case report. Int Med Case Rep J. (2024) 17:909–12. doi: 10.2147/imcrj.S483673. PMID: 39513015 PMC11542473

[3] Xu L Zhao C Bao Y Liu Y Liang Y Wei J . Variation in worldwide incidence of Guillain-Barré syndrome: a population-based study in urban China and existing global evidence. Front Immunol. (2024) 15:1415986. doi: 10.3389/fimmu.2024.1415986. PMID: 39318625 PMC11420027

[4] Leonhard SE Mandarakas MR Gondim FAA Bateman K Ferreira MLB Cornblath DR . Diagnosis and management of Guillain-Barré syndrome in ten steps. Nat Rev Neurol. (2019) 15:671–83. doi: 10.1038/s41582-019-0250-9. PMID: 31541214 PMC6821638

[5] Chen X Ko W Waseem F Cilcic L Kazi N Abdelhafiz A . Guillain-Barré syndrome in older people-a case report and literature review. Dis (Basel Switzerland). (2025) 13:306. doi: 10.3390/diseases13090306. PMID: 41002742 PMC12468067

[6] Shahrizaila N Lehmann HC Kuwabara S . Guillain-barré syndrome. Lancet. (2021) 397:1214–28. doi: 10.1016/s0140-6736(21)00517-1. PMID: 33647239

[7] Ripellino P Schreiner B Latorre D . Expanding our understanding of Guillain-Barré syndrome: recent advances and clinical implications. Eur J Immunol. (2024) 54:e2250336. doi: 10.1002/eji.202250336. PMID: 39188201

[8] Thatikonda N Lerint A Takle C Fang X Patel C . Albuminocytologic dissociation and the impact of age-adjusted cerebrospinal fluid protein levels in Guillain-Barré syndrome. Neurol Int. (2025) 17:18. doi: 10.3390/neurolint17020018. PMID: 39997649 PMC11858027

[9] Bellanti R Rinaldi S . Guillain-Barré syndrome: a comprehensive review. Eur J Neurol. (2024) 31:e16365. doi: 10.1111/ene.16365. PMID: 38813755 PMC11235944

[10] Ahsan A Ibrahim O Ayesha M Hassni AA Saif S Mahin FE . Fusion of molecular mimicry, epigenetic predisposition, and new onset GBS: a narrative review of current understanding and future directions. Ann Med Surg (2012). (2026) 88:1532–40. doi: 10.1097/ms9.0000000000004612. PMID: 41675728 PMC12889248

[11] Yuki N Hartung HP . Guillain-barré syndrome. N Engl J Med. (2012) 366:2294–304. doi: 10.1056/NEJMra1114525. PMID: 22694000

[12] Wakerley BR Uncini A Yuki N . Guillain-Barré and Miller Fisher syndromes--new diagnostic classification. Nat Rev Neurol. (2014) 10:537–44. doi: 10.1038/nrneurol.2014.138. PMID: 25072194

[13] Silva CA Cocuzza M Carvalho JF Bonfá E . Diagnosis and classification of autoimmune orchitis. Autoimmun Rev. (2014) 13:431–4. doi: 10.1016/j.autrev.2014.01.024. PMID: 24424181

[14] Ravindranath MH Muthugounder S Presser N Selvan SR Portoukalian J Brosman S . Gangliosides of organ-confined versus metastatic androgen-receptor-negative prostate cancer. Biochem Biophys Res Commun. (2004) 324:154–65. doi: 10.1016/j.bbrc.2004.09.029. PMID: 15464996

[15] Chakroun S Faucher A Gueguen A Fadlallah J Corvilain E Kubis N . Primary neurolymphomatosis: a literature review. Eur J Neurol. (2025) 32:e70173. doi: 10.1111/ene.70173. PMID: 40265708 PMC12015973

[16] Elendu C Osamuyi EI Afolayan IA Opara NC Chinedu-Anunaso NA Okoro CB . Clinical presentation and symptomatology of Guillain-Barré syndrome: a literature review. Med (Baltim). (2024) 103:e38890. doi: 10.1097/md.0000000000038890. PMID: 39058828 PMC11272278

